# Psychometric Properties of the Coparenting Relationship Scale in Primiparous Mothers and Partners in Karachi, Pakistan

**DOI:** 10.3390/children13101329

**Published:** 2026-09-30

**Authors:** Salima Sulaiman, Aliyah Dosani, Ntonghanwah Forcheh

**Affiliations:** 1 School of Nursing, Faculty of Applied Health Sciences, Brock University, 1812 Sir Isaac Brock Way, St. Catharines, ON L2S 3A1, Canada; 2 School of Nursing and Midwifery, Mount Royal University, 4825 Mount Royal Gate SW, Calgary, AB T3E 6K6, Canada; 3 Department of Community Health Sciences, Cumming School of Medicine, University of Calgary, Calgary, AB T2N 4Z6, Canada; 4 O’Brien Institute of Public Health, University of Calgary, Calgary, AB T2N 4Z6, Canada; 5 School of Nursing, Queens University, 99 University Avenue, Kingston, ON K7L 3N6, Canada; 6 Department of Mathematical Sciences, University of Zululand, Private Bag X1001, KwaDlangezwa, Empangeni 3886, South Africa

**Keywords:** coparenting relationship scale, partners, mothers, psychometric properties, factor analysis

## Abstract

**Highlights:**

**What are the main findings?**

- The CRS supported a seven-factor structure among mothers.
- A preliminary six-factor structure emerged for partners, combining Coparenting Closeness and Endorsement of Partner’s Parenting.

**What are the implications of the main findings?**

- The CRS factor structure may require further evaluation among partners in this cultural context.
- The low reliability of the Division of Labor subscale highlights the need for further psychometric assessment among both mothers and partners.

**Abstract:**

**Background**: The coparenting relationship plays an important role in raising a child. The Coparenting Relationship Scale (CRS) is widely used to assess the multidimensional nature of coparenting; however, its psychometric properties have not been examined separately among mothers and partners, particularly in low- and middle-income country settings. This study evaluated the factor structure of the CRS among first-time mothers and partners in Karachi, Pakistan. **Methods**: The full study recruited primiparous mothers and their partners during the first or second trimester from a tertiary care hospital in Karachi, Pakistan. The analysis utilized data from 120 mothers and 102 partners who consented to participate at 12 weeks postpartum. Confirmatory factor analyses (CFAs) were performed separately for mothers and partners using the WLSMV estimator. Internal consistency was assessed using Cronbach alpha coefficients for each CRS subscale. **Results**: The seven-factor model demonstrated good model fit among mothers, with latent factor correlations within the admissible range and standardized factor loadings ranging from 0.49 to 0.92. Among partners, preliminary findings indicated that a six-factor model in which Coparenting Closeness and Endorsement of Partner’s Parenting were combined provided a more appropriate representation of the data. Most CRS subscales demonstrated satisfactory internal consistency; however, the Division of Labor subscale demonstrated low reliability among both mothers and partners. **Conclusions**: The findings provide preliminary evidence regarding the factor structure of the CRS among Pakistani mothers and partners.

## 1. Introduction

The coparenting relationship is defined as the joint parenting of an infant and refers to the ways in which women and partners collaborate, coordinate, and provide mutual support in fulfilling their parenting roles [1]. In this study, the term partner refers to the father of the child born to a mother recruited into the study. Coparenting plays a central role in parental and child well-being [2,3,4]. For example, supportive coparenting is positively associated with children’s emotion regulation, mental health, self-esteem, and adjustment, including internalizing and externalizing behaviours [3,4,5,6,7,8]. Collaborative coparenting enables parents to share responsibilities, manage stress more effectively, foster confidence in their parenting abilities [1,4], and lower the risk of parental burnout [9]. In contrast, conflictual or unsupportive coparenting increases parental stress [10,11], reduces responsiveness to partners’ needs and feelings, and creates frustration when working together on child-related goals and decision-making [12], which in turn diminishes parenting confidence [13]. Taken together, the quality of the coparenting relationship profoundly shapes the overall parenting environment and long-term family well-being.

The Coparenting Relationship Scale (CRS) is a common tool that measures coparenting relationship quality [14]. The original CRS is comprised of seven subscales [14] grounded in Feinberg’s four-domain coparenting framework: childrearing agreement, coparental support/undermining, division of labor, and joint management of family dynamics [1]. The childrearing agreement domain is assessed through the Childrearing Agreement subscale, measuring the extent to which parents agree on parenting values, standards, and practices [14]. The coparental support/undermining domain is represented by three subscales: Coparenting Support, Endorsement of Partner’s Parenting, and Coparenting Undermining [14]. Coparenting support assesses perception of support received from their partner; whereas the endorsement of partner’s parenting evaluates one’s own positive attitude toward the partner’s parenting [14], and coparenting undermining gauges the perception of negative interactions such as being criticized or blamed by their partner regarding their parenting choices [14]. The joint management of family dynamics domain is measured using the Exposure to Conflict subscale, which assess the extent to which parents expose their children to coparental conflict [14].The division of labor domain is assessed through the Division of Labor subscale, which measures satisfaction with the partner’s contributions to childrearing responsibilities [14]. In addition, the CRS includes a subscale assessing the extent to which coparenting enhances intimacy and strengthens the couple’s relationship, referred to as Coparenting Closeness, which assesses the extent to which coparenting enhances intimacy and strengthens the couple’s relationship [14]. Although some psychometric studies have reported variations in the factor structures across populations, the original seven-factor structure was retained as the hypothesized model and empirically tested using a sample of Pakistani first-time mothers and their partners.

Since its development, the CRS has been validated across multiple languages and countries, demonstrating varying factor structures. A multi-country study, comprising 15 datasets across 10 countries and nine languages, identified a consistent six-factor, 33-item structure across diverse contexts [15]; however, the division of labor subscale was excluded from the analysis. Notably, the majority of datasets were drawn from high-income countries, with only two studies conducted in upper-middle-income settings. Most datasets included parents of children in early to middle childhood, with only four datasets including parents of infants under one year of age, and only 10 datasets including data from both parents.

At the country level, variations in factor structure have been reported, including a six-factor, 27-item model among Chinese couples [16], a four-factor, 35-item model among Brazilian couples [17], a two-factor, 20-item model among Spanish couples [18], a seven-factor, 37-item model among Italian couples [19], and a six-factor, 28-item model among Romanian couples [20]. These differences may be explained by variations in participant characteristics. For example, the age of children varied across studies, ranging from elementary school-aged children [16,18,19], to 0–36 years [20], and 4–6 years [17], while marital status varied from exclusively married couples [16,17,19] to more diverse family structures, including divorced parents and stepfamilies [18,20]. In addition, parents in all studies had more than one child, and the mean age of the parents ranged from 36.6 to 57 years. This combined analysis of mothers and partners across varied child ages and family compositions implicitly assumes that the scale functions similarly across genders and among parents with diverse characteristics. However, this assumption may overlook meaningful differences in how mothers and partners perceive and enact coparenting, potentially obscuring gender-specific patterns in beliefs, responsibilities, and lived experiences. Furthermore, the variability in factor structures raises concerns regarding the cross-cultural stability and conceptual equivalence of the CRS, suggesting that the underlying constructs of coparenting may not be uniformly represented across cultural contexts. These gaps highlight the need for further psychometric research that examines the CRS separately among mothers and partners and within more homogeneous family and child-developmental contexts. Establishing a reliable and valid factor structure within specific parent and cultural groups is essential to ensure that the CRS accurately captures coparenting experiences and can be used in research and practice.

Furthermore, only two studies that examined the psychometric properties of the CRS specifically among mothers found that the original seven-factor model performed poorly in this group [21,22]. The mothers included in these studies had at least one child under 18 years of age [21] and were raising an average of two children [22]. When mothers have multiple children, parenting experiences and responses may vary according to the age, developmental stage, and needs of each child. For example, parenting a school-aged child may involve different demands and experiences than parenting an infant. Consequently, responses may be influenced by child-related factors. In contrast, the present study focused exclusively on primiparous mothers, thereby reducing potential confounding factors related to differences in parenting experiences across multiple children of different ages and developmental stages. This represents an important methodological strength of the study. Importantly, no known studies to date have examined the factor structure of the CRS exclusively among partners. This represents a significant gap, as they may interpret coparenting constructs differently due to distinct sociocultural roles and expectations [23]. There is therefore a clear need for psychometric research that evaluates the CRS separately among mothers and partners. Separate validation is critical to determine whether the CRS adequately captures the distinct experiences and perceptions of mothers and of partners, and to establish whether its underlying factor structure is appropriate for each parent group. This is particularly important among first-time parents, for whom coparenting is developing within the context of a new family transition and where there is no ambiguity regarding the child being coparented.

Coparenting processes may be shaped by sociocultural norms, parental beliefs, and contextual expectations, which can influence how mothers and partners engage in parenting [1], particularly across diverse contexts including low- and middle-income countries (LMIC). For example, in Pakistan, some evidence suggests that partners may place greater emphasis on financial provision, while mothers may assume a greater share of daily childcare, feeding, and household tasks [23]. Partners may also participate in activities that involve mutual decision-making such as breastfeeding support, healthy food choices, teaching, stimulation activities, and health-seeking practices for the child [23,24]. Evidence further suggests that partners may provide support when mothers are overburdened or occupied with other responsibilities, including soothing infants and providing routine caregiving [23,24,25] and engaging in meaningful interaction and play with their children [24]. These patterns suggest that coparenting roles and responsibilities may vary according to sociocultural and family context and may influence how coparenting relationship are experienced and understood in LMIC contexts. Recognizing these contextual influences is therefore important when examining the coparenting relationship and evaluating whether a measure of coparenting functions similarly across cultural settings [15]. Guided by this rationale, the objective of this study was to evaluate the original seven-factor structure of the CRS separately among primiparous mothers and partners in Karachi, Pakistan, including an examination of factor loadings, inter-factor correlations, and internal consistency. Alternative factor structures will also be considered if the initially hypothesized seven-factor structure is not adequately supported by the data.

## 2. Materials and Methods

### 2.1. Participants

Eligible participants were women who were pregnant for the first time with a singleton infant and were in the first or second trimester of pregnancy at the time of recruitment. Eligible male participants were partners of the enrolled women. For this study, inclusion was restricted to participants who completed the baseline assessment, consented to participate in the 12-weeks postpartum assessment, and had a live-born infant. Detailed inclusion and exclusion criteria for the full study have been reported elsewhere [26]. Recruitment was conducted at a tertiary care hospital in Karachi, Pakistan, between September 2018 and February 2020. The analytic sample comprised 120 first-time mothers and 102 partners. Because the psychometric properties of the CRS were evaluated separately among mothers and partners, all participants were included in the analyses to maximize statistical power, instead of restricting to a 101 complete couple-wise sample.

### 2.2. Ethical Consideration of Data Collection

The study was conducted in accordance with the Declaration of Helsinki, and the protocol was approved by the Aga Khan University Hospital, University of Toronto, Brock University, and Mount Royal University. Written informed consent was obtained from all participants.

### 2.3. Data Collection and Measures

A research assistant contacted participants over the telephone who had consented at the baseline to participate in the 12-weeks postpartum assessment. The English version of the original 35-item CRS questionnaire was completed independently by the mothers and partners. The CRS [14] is a previously validated 35-item self-report questionnaire that measures how each partner perceives the coparenting relationship with their partner. All 35 items, including the two Division of Labor items, were retained to allow for an evaluation of the original CRS factor structure in Pakistani mothers and partners. The instrument was pilot tested with 10 couples attending a community health clinic, and no modifications were required.

The CRS was developed as a multidimensional measure of coparenting, and the 35 items are organized into seven subscales: Childrearing Agreement (4 items), Coparenting Support (6 items), Coparenting Undermining (6 items), Endorsement of Partner’s Parenting (7 items), Division of Labor (2 items), Exposure to Conflict (5 items), and Coparenting Closeness (5 items) [14]. The CRS tool has demonstrated good internal consistency, ranging from 0.91 to 0.94 among mothers and partners [14]. Items are rated on a seven-point response scale ranging from 0 to 6. For six subscales, excluding Exposure to Conflict, responses range from 0 = “that is not true of us” to 6 = “very true of us”. For the Exposure to Conflict subscale, respondents indicate the frequency with which conflict occurs when they are physically present with their partner and child, with responses ranging from 0 = “never” to 6 = “several times a week”.

### 2.4. Data Analysis

Descriptive statistics, including means, standard deviations, and floor and ceiling effects were computed for participant characteristics and CRS item responses,. Floor or ceiling effects exceeding 15% were considered indicative of substantial response asymmetry [27], supporting the use of polychoric correlations in confirmatory factor analysis (CFA) rather than traditional Pearson correlation matrices [28,29]. Internal consistency reliability was assessed using Cronbach’s alpha coefficients for each subscale. To evaluate the hypothesized seven-factor structure of the CRS, confirmatory factor analysis was conducted using the weighted least squares mean and variance (WLSMV) estimator applied to polychoric correlation matrices, which is appropriate for ordinal response data exhibiting substantial floor and ceiling effects. Model fit was evaluated using the comparative fit index (CFI), Tucker–Lewis index (TLI), root mean square error of approximation (RMSEA), and standardized root mean square residual (SRMR). Construct validity and the distinctiveness of the proposed dimensions were assessed using standardized factor loadings, reliability coefficients, and latent factor correlations, inspection of modification indices (mi), and standardized expected parameter change (SEPC.all) values to identify areas of model misfit. Separate CFA measurement models corresponding to each of the seven CRS subscales were first fitted to determine the adequacy of each proposed dimension. A full seven-factor model using all 35 items, each loaded on their respective theoretical domain was subsequently fitted to evaluate the overall factor structure of the instrument. Where indicated by the CFA results, theoretically justifiable model modifications and alternative factor structures were evaluated and compared. All analyses were conducted in R version 4.4.3 (R Core Team, 2025). Confirmatory factor model parameters and required polychoric correlation matrices were estimated using the lavaan package (version 0.6-21).

## 3. Results

### 3.1. Participants

The mean age of mothers was 26.7 years (SD = 3.09). Mothers represented diverse ethnic backgrounds, including Muhajir (35%), Punjabi (15.8%), Sindhi (10%), Gujarati (9.2%), Memon (5.8%), Pathan (4.2%), Katchi (4.2%), and other ethnicities (15.8%). Half of the mothers (50%) were housewives, and more than 94% had completed a baccalaureate or graduate degree. The mean age of the partners was 30.6 years (SD = 3.36). Partners were also ethnically diverse, with Muhajir (33.3%), Punjabi (16.7%), Sindhi (16.7%), Gujarati (10.8%), Memon (4.9%), Katchi (3.9%), and other ethnicities (13.7%). Over 95% of partners had a baccalaureate or graduate degree. All couples were married.

### 3.2. Preliminary Analyses

Means, standard deviations, floor and ceiling effects, and polychoric inter-item correlation matrices for mothers and partners are reported (see Supplementary Tables S1 and S2). Polychoric item correlations ranged from −0.63 to 0.86 among mothers and from −0.72 to 0.93 among partners. Several items representing positive dimensions of coparenting, including endorsement of partner’s parenting, coparental support, and coparenting closeness, demonstrated high mean scores (mothers: M = 4.4–5.6, SD = 1.0–2.0; partners: M = 4.8–5.7, SD = 0.7–2.1) and substantial ceiling effects. In contrast, items measuring coparenting undermining and exposure to conflict exhibited low mean scores (mothers: M = 0.5–1.7, SD = 1.2–2.1; partners: M = 0.5–1.9, SD = 1.1–2.2) and marked floor effects. Among the mothers, 19 items had ceiling effects exceeding 50%, with between 54.2% and 78.3% of respondents selecting the highest possible response (score = 6). Eleven items for mothers and ten items for partners showed flow effects above 50%, with 50.8–77.5% of respondents selecting the lowest possible response (score = 0). These floor and ceiling effects substantially exceeded the prespecified 15% threshold and support the use of polychoric rather than Pearson correlation matrices in subsequent factor analyses. Collectively, these distributions indicate that positive coparenting behaviours were frequently endorsed, whereas negative coparenting behaviours and conflict-related experiences were relatively uncommon among both mothers and partners.

### 3.3. Confirmatory Factor Analysis of Individual CRS Subscales

Confirmatory factor analysis was performed to evaluate the assumed unidimensional structure of each CRS subscale separately. One-factor CFA models were estimated for all subscales except Division of Labor, which only had two items. The unidimensional structure is presented in Table 1. Given the data limitations of the Division of Labor subscale, its psychometric properties were evaluated using the association between the two items and internal consistency reliability.

#### 3.3.1. Mothers Coparenting Relationship Scale

The separate one-factor CFA model fits for each of the six estimable CRS subscales are summarized in Table 1. Overall, the results provided strong support for the unidimensional structure of the proposed CRS dimensions among mothers. The Childrearing Agreement, Coparenting Closeness, Coparenting Undermining, and Endorsement of Partner’s Parenting subscales demonstrated excellent fit (CFI = 1.00, TLI = 1.00–1.03, RMSEA = 0.00, SRMR = 0.04–0.05). Exposure to Conflict and Coparental Support demonstrated good fits, with CFI and TLI values of 0.99 and SRMR values of 0.06 and 0.08, respectively, although their RMSEA values (0.09 and 0.10) were slightly higher than conventional thresholds. Since the Division of Labor subscale could not be evaluated using a one-factor CFA because it consisted of only two indicators and was therefore under-identified, evidence regarding this dimension was based on the association between its two items (Spearman’s ρ = 0.30; polychoric r = 0.39) and internal consistency reliability (Cronbach’s α = 0.41).

Standardized factor loadings ranged from 0.44 to 0.96 (see Supplementary Table S3). The strongest factor loadings were observed for items in the Exposure to Conflict subscale, where four out of the five items loaded at 0.80 or higher (λ = 0.80–0.84), and for the Coparental Support subscale (λ = 0.68–0.92), including two items with factor loadings exceeding 0.85. Strong factor loadings were also observed for several Endorsement of Partner’s Parenting items (λ = 0.88–0.96). Relatively weak factor loadings (implying low associations with latent measure) were observed for CRS06 (λ = 0.44), CRS07 (λ = 0.46), CRS29 (λ = 0.44), CRS08 (λ = 0.48), and CRS21 (λ = 0.48).

#### 3.3.2. Partners Coparenting Relationship Scale

The results in Table 2 (partners) generally support the unidimensional structure of the proposed CRS dimensions among partners. The Childrearing Agreement, Coparenting Closeness, and Coparenting Support subscales demonstrated excellent fit with CFI and TFI values of 1.00, RMSEA values of 0.00, and SRMR values ranging from 0.01 to 0.07. Coparenting Undermining and Endorsement of Partner’s Parenting demonstrated very good fit, with CFI and TLI values of at least 0.98, RMSEA values between 0.05 and 0.07, and SRMR values between 0.07 and 0.09. Exposure to Conflict demonstrated strong fit, with CFI (1.00), TLI (1.00), and SRMR (0.06). However, its RMSEA = 0.12 was higher than conventional thresholds of 0.08. For the Division of Labor subscale, the two items had moderate Spearman correlation (ρ = 0.46), polychoric correlation (r = 0.61), and internal consistency reliability (Cronbach’s α = 0.56).

Standardized factor loadings ranged from 0.30 to 1.00 (see Supplementary Table S3). The strongest factor loadings were observed for the Exposure to Conflict subscale, where all five items loaded strongly on the latent factor (λ = 0.76–0.97), including three items with factor loadings exceeding 0.90. Similarly, the Coparental Support subscale demonstrated consistently strong factor loadings with three items loading at 0.87–1.00. The few items that showed weak factor loadings were CRS06 (λ = 0.30), CRS28 (λ = 0.41), CRS14 (λ = 0.42), and CRS03 (λ = 0.46).

### 3.4. Confirmatory Factor Analysis of the Full Seven-Factor Coparenting Relationship Scale Model

Although the individual subscale CFA generally demonstrated acceptable to excellent fit as discussed in Section 3.3, several findings, including weaker factor loadings for CRS06, CRS14, CRS03, CRS28, CRS07, CRS29, CRS08, and CRS21, and the comparatively low reliability of the Division of Labor subscale, necessitated further evaluation using the complete CRS measurement model. Accordingly, a seven-factor CFA in which all 35 items were simultaneously specified to load on their respective theoretical domains was fitted. The full model was evaluated separately for mothers and partners to assess the overall factor structure validity of the CRS and the distinctiveness of the proposed coparenting dimensions within each group. Fit indices for the seven-factor CFA models among mothers and partners are presented in Table 2. Additional model diagnostics are presented in supplementary results. These include internal consistency estimates using Cronbach’s α and McDonald’s ω (Supplementary Table S7), convergent validity using AVE (Supplementary Table S8) and discriminant validity based on HTMT (Supplementary Table S9).

#### 3.4.1. Full Seven-Factor Confirmatory Factor Analysis Model for Mothers

The fit indices for mothers suggest an overall very good fit (see Table 2). In particular, CFI = 0.99 and TLI = 0.99 are indicative of an excellent fit among mothers, while the RMSEA = 0.06, 95% CI [0.04–0.06] indicate a good fit, although SRMR = 0.098 is slightly higher than the conventional threshold of 0.08. The results suggest that the 35 items are adequately represented by the seven theoretical CRS factors when evaluated simultaneously. Standardized factor loadings for the full seven-factor model (see Supplementary Table S4) ranged from 0.49 (CRS05) to 0.92 (CRS01). The highest factor loadings were observed for Endorsement of Partner’s Parenting item CRS01 (λ = 0.92), Coparenting Closeness item CRS24 (λ = 0.90), Exposure to Conflict items CRS32-CRS35 (λ = 0.76–0.86), and Coparental Support items CRS25-CRS27 (λ = 0.80–0.87), reflecting strong associations with their respective latent constructs. The only item with a factor loading below 0.50 was CRS05 (λ = 0.49) from the Division of Labor subscale. Although items CRS08 (λ = 0.59), CRS12 (λ = 0.56), CRS21 (λ = 0.57), and CRS29 (λ = 0.55) had comparatively weaker factor loadings, their values were still acceptably large.

Items that exhibited low factor loadings in the individual subscale CFA had substantially larger factor loadings within their latent constructs when evaluated within the full seven-factor model, implying stronger associations. For example, the factor loading for CRS06 increased from 0.44 to 0.72, while factor loadings for CRS07, CRS29, CRS08, and CRS21 increased from 0.46, 0.44, 0.48, and 0.48 to 0.61, 0.55, 0.59, and 0.57, respectively. Hence the performance of these items was better represented when the multidimensional nature of the CRS was explicitly modelled. Furthermore, the full seven-factor model permitted estimation of the Division of Labor subscale, which was not possible in the single-factor modelling framework. Both items of Division of Labor (CRS05: λ = 0.49; CRS20: λ = 0.78) loaded positively, supporting retention of the factor in the model.

Latent factor correlations are presented in Table 3. Latent factor correlations ranged from 0.27 to 0.91 of absolute magnitude. The strongest positive latent factor correlations were observed between Endorsement of Partner’s Parenting and Coparenting Closeness (r = 0.91), Coparental Support and Coparenting Closeness (r = 0.84), and Coparental Support and Endorsement of Partner’s Parenting (r = 0.83). Similarly, strong negative latent factor correlations were observed between Coparental Support and Coparenting Undermining (r = −0.85) and Childrearing Agreement and Coparenting Undermining (r = −0.82). In contrast, Division of Labor demonstrated comparatively weaker associations with the remaining dimensions, ranging from r = 0.27 (Childrearing Agreement) to r = 0.62 (Endorsement of Partner’s Parenting) and from r = −0.49 (Coparenting Conflict) to r = −0.74 (Coparenting Undermining). The two negative coparenting dimensions, Coparenting Conflict and Coparenting Undermining were positively correlated (r = 0.72), while all five positive dimensions were positively correlated with each other, and negatively correlated with each of the negative dimensions.

#### 3.4.2. Full Seven-Factor Confirmatory Factor Analysis Model for Partners

The fit indices for partners indicate an overall acceptable fit of the seven-factor model (see Table 2). The incremental fit indices were high (CFI = 0.98; TLI = 0.98), providing preliminary support for the theoretical multidimensional structure of the CRS. The RMSEA = 0.09, 95% CI [0.08–0.10] suggested reasonable approximate fit, while the SRMR of 0.12 was above the conventional threshold of 0.08. When considered together, the fit indices provide preliminary evidence for the proposed seven-factor structure among partners, although the RMSEA and SRMR values suggest some degree of misfit, and warranted further evaluation of model admissibility and discriminant validity.

As shown in Table 3, two latent factor correlations exceeded the theoretical upper bound of 1.00: Coparenting Closeness—Coparental Support (r = 1.02) and Coparenting Closeness—Endorsement of Partner’s Parenting (r = 1.07). Further diagnostic investigation revealed a negative minimum eigenvalue of the latent factor correlation matrix, indicating linear dependence among some dimensions. In addition, MI and corresponding SEPC.all values were exceptionally large (see Supplementary Table S5), indicating substantial overlap among the Coparenting Closeness, Coparental Support, and Endorsement of Partner’s Parenting dimensions. This was most evident for items CRS02 (MI = 2819.89 and 1238.57), CRS24 (MI = 161.56 and 105.06), CRS01 (MI = 125.23), CRS03 (MI = 106.01), and CRS06 (MI = 72.48, 69.72, and 66.70). Collectively, these findings suggest that the Coparenting Closeness, Coparental Support, and Endorsement of Partner’s Parenting constructs may not be fully distinguishable among partners in this sample. Furthermore, these dimensions appeared to account for the linear dependence among factors that resulted in the non-positive definite latent correlation matrix.

Examination of the remaining admissible latent factor correlations revealed substantial associations among several of the theoretical CRS dimensions based on factor loading values. The strongest positive associations were observed between Coparental Support and Endorsement of Partner’s Parenting (r = 0.85), Childrearing Agreement and Endorsement of Partner Parenting (r = 0.73), and Division of Labor and Endorsement of Partner’s Parenting (r = 0.73). Strong negative associations were observed between Endorsement of Partner’s Parenting and Coparenting Undermining (r = −0.84) and Childrearing Agreement and Coparenting Undermining (r = −0.76). The two negative coparenting dimensions, Exposure to Conflict and Coparenting Undermining, were positively correlated (r = 0.61). The positive coparenting dimensions were positively correlated with one another and negatively correlated with the negative coparenting dimensions.

#### 3.4.3. Follow-Up Evaluation of the Partner Factor Structure

Given the evidence of substantial overlap between the Coparenting Closeness and Endorsement of Partner’s Parenting dimensions, including latent factor correlations exceeding the admissible upper bound of 1.00 and a non-positive definite latent factor correlation matrix, an alternative six-factor CFA model was evaluated. The revised model combined the Coparenting Closeness and Endorsement of Partner’s Parenting subscales into a single latent factor, while retaining the remaining five CRS dimensions unchanged.

The revised model yielded an admissible latent factor correlation matrix, with all latent factor correlations falling within the theoretical bounds (|r| < 1.0) and all eigenvalues being positive (minimum eigenvalue = 0.01). The strongest latent factor correlation was observed between the combined Coparenting Closeness–Endorsement of Partner’s Parenting factor (r = 0.94). Although there was no improvement in the overall fit over the original seven-factor model (CFI = 0.98, TLI = 0.98, RMSEA = 0.09, SRMR = 0.12), reliability analysis of the combined Coparenting Closeness–Endorsement of Partner’s Parenting subscale yielded a Cronbach’s α = 0.75; standardized α = 0.79, which represented a marked improvement over the reliabilities of the separate Coparenting Closeness (α = 0.56) and Endorsement of Partner’s Parenting (α = 0.59) subscales (see Table 4). Together with the evidence from the latent factor correlations, eigenvalue diagnostics, and MI, these results suggest that perceptions of Coparenting Closeness and Endorsement of Partner’s Parenting were not empirically indistinguishable among partners in this sample and may be more appropriately represented as a single dimension.

### 3.5. Summary and Comparison of the Seven-Factor Coparenting Relationship Scale Models

Overall, the seven-factor CFA model provided stronger psychometric support for mothers. The model demonstrated good to excellent fit (CFI = 0.99, TLI = 0.99, RMSEA = 0.06), and all latent factor correlations fell within the admissible range. Standardized factor loadings were generally strong, ranging from 0.49 to 0.92, with several items exhibiting stronger factor loadings in the full model than in the individual subscale models. In addition, the largest MI (MI = 52.5) was modest and all |SEPC.all|< 1.00 (see Supplementary Table S6), providing further support for the adequacy of the theoretical seven-factor structure among mothers.

For partners, the full seven factor CFA model yielded preliminary evidence of acceptable fit indices (CFI = 0.98, TLI = 0.98, RMSEA = 0.09) and provided preliminary support for the theoretical seven-factor CRS structure. Factor loadings were generally moderate to strong. Several items demonstrated improved factor loadings compared to when individual subscale models were evaluated. However, latent factor correlations between Coparenting Closeness and Coparenting Support, and Coparenting Closeness and Endorsement of Partner’s Parenting exceeded the admissible upper bound of 1.00, indicating an inadmissible solution. Further diagnostic analyses revealed a non-positive definite latent factor correlation matrix with a negative eigenvalue and several exceptionally MI and SEPC.all values. Taken together, these findings suggest substantial overlap among the Coparenting Closeness, Coparental Support, and Endorsement of Partner’s Parenting dimensions, and indicate that the seven-factor solution did not provide an admissible representation of the partners’ data.

Based on the diagnostic results, a follow-up six-factor CFA model was evaluated in which the Coparenting Closeness and Endorsement of Partner’s Parenting dimensions were combined into a single latent factor. Although the revised model did not improve overall fit (CFI = 0.98, TLI = 0.98, RMSEA = 0.09, SRMR = 0.12), it resolved the inadmissible latent correlation matrix, all latent factor correlations fell within theoretical bounds, and all eigenvalues were positive. In addition, the combined Coparenting Closeness–Endorsement of Partner’s Parenting factor demonstrated substantially improved reliability (α = 0.75; standardized α = 0.79) compared with the separate Coparenting Closeness (α = 0.56) and Endorsement of Partner’s Parenting (α = 0.59) subscales. These findings did not warrant abandonment of the theoretical seven-factor CRS model. Rather, they suggest that Coparenting Closeness and Endorsement of Partner’s Parenting may not represent empirically distinct constructs among partners in this sample and may be more appropriately represented as a single dimension.

### 3.6. Descriptive Statistics for Raw Subscales

Table 5 presents descriptive statistics for the CRS subscales among mothers and partners. Subscale scores were calculated as the average of the constituent items within each dimension, producing scores on a common 0–6 scale, where higher values indicate greater endorsement of the underlying construct. This standardization ensured that descriptive statistics can be compared directly across dimensions despite differences in subscale length.

Among the mothers, the highest mean scores were observed for Coparenting Closeness (M = 5.2, 95% CI: 5.01–5.35), Coparental Support (M = 5.1, 95% CI: 4.89–5.29), and Endorsement of Partner’s Parenting (M = 5.1, 95% CI: 4.96–5.30), whereas Exposure to Conflict (M = 0.9, 95% CI: 0.69–1.08) and Coparenting Undermining (M = 1.2, 95% CI: 0.96–1.37) had the lowest mean scores. A similar pattern was observed among partners, with the highest mean scores recorded for Coparenting Closeness (M = 5.3, 95% CI: 5.11–5.42), Coparental Support (M = 5.2, 95% CI: 5.03–5.37), and Endorsement of Partner’s Parenting (M = 5.2, 95% CI: 5.05–5.36), while Exposure to Conflict (M = 0.8, 95% CI: 0.61–1.05) and Coparenting Undermining (M = 1.5, 95% CI: 1.18–1.72) remained comparatively low. Division of Labor displayed the greatest variability in both groups (SD = 1.73 among mothers; SD = 1.77 among partners) and was the only dimension for which partners reported notably higher average scores than mothers (M = 4.6 vs. 3.7).

## 4. Discussion

This study aimed to examine the factor structure of the CRS for both mothers and partners separately. The 35-items CRS adequately represents the seven CRS factors for mothers. For partners, a preliminary six-factor solution is proposed in which Coparenting Closeness and Endorsement of Partner’s Parenting are combined into a single subscale. Additionally, the Division of Labor subscale was identified as the only subscale that showed variability for both mothers and partners.

In comparing our findings of the mother’s data with the existing literature, several differences emerged. Our findings on mothers’ data confirmed a seven-factor structure, which contrasts with the six-factor models reported in studies conducted with Hungarian and Portuguese mothers [21,22]. Differences in parenting stage of the children and family composition may account for the contrasting CRS factor structure reported in these other studies. Both the Hungarian and Portuguese samples consisted of parents raising multiple children, including families with children under 18 years of age [21,22], meaning that coparenting behaviours may have reflected roles for multiple children, including older and school-aged children who have different needs than a newborn. As such, parenting multiple children of various ages potentially introduces child-specific confounding factors into CRS responses and may blur distinctions among coparenting dimensions [21,22]. In contrast, our sample was comprised of first-time mothers and their partners, all parenting a single 12-week-old infant. This homogeneous, well-educated sample, surveyed during the early postpartum period of their first child, a period characterized by role adjustment, heightened emotional demands, and evolving partner dynamics [1], provided an excellent opportunity to evaluate the hypothesized seven-factor CRS tool and study how coparenting constructs are conceptualized and measured. These contextual differences may contribute to variations in how coparenting dimensions are experienced and reflected in the underlying CRS factor structure. This further highlights the importance of context-specific validation, as presented in this study.

To our knowledge, this is the first study to evaluate the factor structure of the CRS exclusively among partners. Previous psychometric evaluations of the CRS have been conducted using data from couples rather than partners alone [16,20,30]. Our findings support a six-factor structure for partners that is largely consistent with Feinberg’s original CRS, with the exception that the Coparenting Closeness and Endorsement of Partner’s Parenting subscales loaded onto a single factor [14]. The similarity between our findings and Feinberg’s original study [14] may be partly explained by the comparable characteristics of the two samples, as both consisted of heterosexual couples expecting their first child at the time of recruitment. Notably, the Coparenting Closeness and Endorsement of Partner’s Parenting subscales were empirically indistinguishable among partners in our sample, suggesting that these constructs may be more appropriately represented as a single dimension in this cultural context. The Endorsement of Partner’s Parenting subscale assesses a partner’s positive evaluation of his partner’s parenting abilities, whereas Coparenting Closeness reflects the extent to which parenting enhances intimacy and strengthens the couple’s relationship [14]. The convergence of these subscales suggests that partners who expressed greater appreciation of their partner’s parenting may also have experienced greater relational closeness. One possible explanation is that partners in some LMIC contexts may have less involvement in day-to-day childcare than mothers and may be less likely to accompany children to healthcare services because of employment demands and financial responsibilities [31,32]. Partners may also experience pressure to fulfil the provider role and balance work and family responsibilities [33,34]. These factors could potentially reduce opportunities to differentiate between their partner’s parenting competence and the emotional quality of the coparenting relationship. Similarly, broader gender role expectations and patriarchal social structures, such as the expectation of partners to prioritize paid work and financial provision, and women to assume primary responsibility for childrearing and family care, may be relevant to how coparenting and partner support are experienced in some cultural contexts [35]. It is possible that in contexts where mothers assume greater responsibility for childrearing and family care, expressions of support or appreciation for a partner’s parenting may be particularly meaningful and may be associated with greater emotional intimacy and relationship closeness. Consequently, partners’ perceptions of their partner’s parenting and their sense of closeness within the coparenting relationship may become closely intertwined, leading the two subscales to function as a single underlying dimension in this setting. This interpretation should be considered as a hypothesis for future research rather than a conclusion supported by the present data. The observed convergence may therefore reflect the ways in which partners conceptualize parenting support and relational closeness within their specific sociocultural context. Future studies should validate coparenting measures among partners in diverse cultural settings.

Our findings demonstrated satisfactory internal consistency for most CRS subscales (α = 0.66–0.85 for mothers; α = 0.73–0.87 for partners), except for the Division of Labor subscale, which demonstrated low internal consistency in both mothers (α = 0.413) and partners (α = 0.559). This finding is consistent with previous validations of the CRS in the American [14], Portuguese [21], and French [36] contexts, where the Division of Labor subscale also demonstrated comparatively lower reliability. More broadly, the psychometric performance of this subscale has been inconsistent across cultural contexts. For example, a previous meta-analysis found that a six-factor structure excluding Division of Labor provided an adequate fit across data from 10 countries [15]. In contrast, validation studies conducted in Romania and France supported Division of Labor as a distinct and meaningful dimension of coparenting [19,36]. These mixed findings raise questions about the conceptual consistency of this subscale and whether it captures a universal dimension of coparenting across diverse cultural settings. One possible explanation is that perceptions of the division of caregiving responsibilities may be influenced by cultural norms and gender-role expectations, particularly in contexts where parenting responsibilities may be more strongly gendered. In some LMIC contexts, mothers may be more likely to assume primary responsibility for infant care, including breastfeeding, feeding, diapering, and other daily childcare tasks, whereas partners may be more likely to assume financial provider responsibilities and participate selectively in activities such as play and recreation [23]. If such patterns characterize the experiences of families in these settings, responses to Division of Labor items may reflect culturally shaped expectations regarding parental roles rather than the actual distribution of caregiving responsibilities. This could potentially explain why this subscale showed weaker associations with other dimensions of coparenting in our context. Future research should examine the conceptual relevance, cultural sensitivity, and measurement invariance of the Division of Labor subscale across diverse cultural and socioeconomic contexts before assuming that it functions equivalently across populations.

### 4.1. Limitations

Several limitations should be considered when interpreting these findings. The recruitment of first-time mothers during early pregnancy and their partners, with both partners required to consent to participation and remain in the study through 12 weeks postpartum, may have preferentially attracted couples with a relatively high socioeconomic status. For example, all parents in our study completed the survey in English, had at least a university-level education, and the partners were employed in stable positions within the government or private sectors. The sample therefore consisted of English-speaking, highly educated, married, first-time parents recruited from a single tertiary-care hospital in Karachi, resulting in a relatively homogeneous sample. These characteristics may limit the representativeness of the findings and should be considered when interpreting their applicability to the broader population of Pakistani parents. At the same time, these eligibility criteria, combined with the fact that relatively few pregnant women attend prenatal clinics with their partners, restricted the pool of eligible participants. For example, enrolling up to 120 mothers along with 101 partners during early pregnancy, with both partners consenting to remain in the study through 12 weeks postpartum, required access to a large population of pregnant women. The relatively modest sample size should be interpreted from this context.

The CRS also demonstrated marked floor and ceiling effects, with several items showing more than 50% of responses concentrated at the extreme response categories. This restricted variability may limit scale sensitivity and result in sparse response distributions, potentially affecting the stability of polychoric correlations and the interpretation of the preliminary factor-analytic findings. The six-factor partner solution was developed and tested within the same small sample and should therefore be considered as a tentative, sample-specific solution requiring independent replication rather than an established alternative factor structure. Furthermore, the study relied on a relatively small sample, which reduces statistical power and constrains the extent to which the results can be applied to broader populations. In addition, the parents who participated may represent a more supportive and engaged subset, as both parents were present in the clinic at the time of recruitment and consented to take part in the study, suggesting that the present findings may be more reflective of highly engaged individuals than of the wider population of expectant parents in Pakistan.

### 4.2. Implications

The sample consisted of first-time mothers and partners who were predominantly married, highly educated, and from diverse ethnic backgrounds. Parents with these characteristics may differ in their coparenting experiences compared to those who are unmarried, have lower levels of education, have multiple children, or are living in different sociocultural and economic contexts. Therefore, future studies should include larger and more heterogeneous samples drawn from multiple regions, socioeconomic strata, educational levels, and linguistic groups to strengthen the validity and generalizability of the scale.

In addition, future research should further examine culturally grounded factors that may account for the differences observed in the factor structure of the CRS. Cultural norms, gender role expectations, family systems, and socioeconomic conditions may all influence how coparenting is experienced, understood, and expressed. These contextual factors may shape the emergence, interpretation, and stability of specific constructs across diverse populations, particularly within varied cultural settings.

## 5. Conclusions

Our study is the first to examine the factor structure of the CRS separately among mothers and partners in the Pakistani context. The findings confirm the theoretical seven-factor structure for mothers and a modified six-factor structure for partners, with the latter emerging after combining the Coparenting Closeness and Endorsement of Partner Parenting subscales. Although enrolling only primiparous mothers in early pregnancy and their partners who consented to follow-up through 12 weeks postpartum restricted the target population size, this approach ensured a homogenous sample, producing a more reliable model fit than is possible with heterogenous samples of similar sizes. Variability was also observed in the Division of Labor subscale, suggesting that the conceptualization of this subscale may differ across cultural contexts. These findings highlight the importance of considering sociocultural and family-contextual factors when evaluating the coparenting relationship in Pakistan and support the need for further validation of the CRS across diverse Pakistani populations.

## Figures and Tables

**Table 1:** One-factor CFA fit statistics for Coparenting Relationship Scale subscales.

Subscale	χ^2^	Df	CFI	TLI	RMSEA	SRMR
Mothers						
Childrearing Agreement	1.48	2	1.00	1.01	0.00	0.04
Coparenting Closeness	3.03	5	1.00	1.01	0.00	0.04
Exposure of Conflict	10.21	5	0.99	0.99	0.09	0.06
Coparental Support	18.71	9	0.99	0.99	0.10	0.08
Coparenting Undermining	3.98	9	1.00	1.03	0.00	0.04
Endorsement of Partner’s Parenting	8.74	14	1.00	1.01	0.00	0.05
Division of Labor						
Partners						
Childrearing Agreement	0.18	2	1.00	1.01	0.00	0.01
Coparenting Closeness	4.34	5	1.00	1.00	0.00	0.07
Exposure to Conflict	11.64	5	1.00	1.00	0.12	0.06
Coparental Support	7.23	9	1.00	1.00	0.00	0.06
Coparenting Undermining	13.34	9	0.99	0.99	0.07	0.07
Endorsement of Partner’s Parenting	17.34	14	0.99	0.98	0.05	0.09
Division of Labor						

Note. χ^2^ = chi-square model fit; df = degrees of freedom; CFI = Comparative Fit Index; TLI = Tucker–Lewis Index; RMSEA = Root Mean Square Error of Approximation; SRMR = Standardized Root Mean Square Residual. Models were estimated using the weighted least squares mean and variance adjusted (WLSMV) estimator. The Division of Labor subscale contains only two items and therefore does not yield an identified one-factor CFA model; fit statistics are not reported.

**Table 2:** Confirmatory factor analysis fit indices for the seven-factor Coparenting Relationship Scale model among mothers and partners.

Group	Chi-Sq	DF	CFI	TLI	RMSEA	95% CI	SRMR
Mother	782.97	539	0.99	0.99	0.06	0.04, 0.06	0.01
Partner	854.00	539	0.98	0.98	0.09	0.08, 0.10	0.12

Note. WLSMV estimator used throughout. CFI and TLI values ≥ 0.95 indicate good fit, RMSEA ≤ 0.06 indicates close fit, and SRMR ≤ 0.08 indicates acceptable fit; CI, Confidence Interval.

**Table 3:** Latent factor correlations.

	Childrearing Agreement	Coparenting Closeness	Exposure to Conflict	Coparental Support	Coparenting Undermining	Endorsement of Partners Parenting	Division of Labor
Mothers							
Childrearing Agreement	1.00						
Coparenting Closeness	0.52	1.00					
Exposure to Conflict	−0.48	−0.62	1.00				
Coparenting Support	0.68	0.84	−0.69	1.00			
Coparenting Undermining	−0.82	−0.71	0.72	−0.85	1.00		
Endorsement of Partners Parenting	0.54	0.91	−0.73	0.83	−0.69	1.00	
Division of Labor	0.27	0.41	−0.49	0.46	−0.74	0.62	1.00
Partners							
Childrearing Agreement	1.00						
Coparenting Closeness	0.69	1.00					
Exposure to Conflict	−0.61	−0.65	1.00				
Coparental Support	0.50	1.02 *	−0.54	1.00			
Coparenting Undermining	−0.76	−0.75	0.61	−0.58	1.00		
Endorsement of Partners Parenting	0.73	1.07 *	−0.64	0.85	−0.84	1.00	
Division of Labor	0.60	0.70	−0.62	0.49	−0.74	0.73	1.00

* Inadmissible correlation—requires further diagnostic investigation.

**Table 4:** Sample reliability coefficients for Coparenting Relationship Scale subscales.

Subscales	Items	Alpha	Mean Inter Item Correlations
Mothers			
Childrearing Agreement	4	0.704	0.494
Coparenting Closeness	5	0.713	0.504
Exposure to Conflict	5	0.82	0.617
Coparental Support	6	0.849	0.644
Coparenting Undermining	6	0.664	0.406
Endorsement of Partner Parenting	7	0.729	0.484
Division of Labor	2	0.413	0.272
Partners			
Childrearing Agreement	4	0.738	0.536
Coparenting Closeness	5	0.562	0.354
Exposure to Conflict	5	0.877	0.722
Coparental Support	6	0.784	0.555
Coparenting Undermining	6	0.786	0.543
Endorsement of Partner Parenting	7	0.587	0.35
Division of Labor	2	0.559	0.414
Coparenting Closeness–Endorsement of Partner Parenting		0.75	0.240

**Table 5:** Descriptive statistics for the Coparenting Relationship Scale subscales among mothers and partners.

	Subscales	Mean	95% CI	SD	Median	Min	Max
Mothers							
1	Childrearing Agreement	4.5	4.23, 4.73	1.39	4.8	0.0	6.0
2	Coparenting Closeness	5.2	5.01, 5.35	0.94	5.4	0.0	6.0
3	Exposure to Conflict	0.9	0.69, 1.08	1.08	0.6	0.0	6.0
4	Coparental Support	5.1	4.89, 5.29	1.12	5.5	0.0	6.0
5	Coparenting Undermining	1.2	0.96, 1.37	1.13	1.0	0.0	5.5
6	Endorsement of Partner’s Parenting	5.1	4.96, 5.30	0.95	5.3	0.0	6.0
7	Division of Labor	3.7	3.40, 4.02	1.73	3.5	0.0	6.0
Partners							
1	Childrearing Agreement	4.4	4.07, 4.63	1.42	4.5	1.5	6.0
2	Coparenting Closeness	5.3	5.11, 5.42	0.79	5.4	3.2	6.0
3	Exposure to Conflict	0.8	0.61, 1.05	1.13	0.4	0.0	4.6
4	Coparental Support	5.2	5.03, 5.37	0.89	5.5	1.7	6.0
5	Coparenting Undermining	1.5	1.18, 1.72	1.38	1.0	0.0	5.0
6	Endorsement of Partner’s Parenting	5.2	5.05, 5.36	0.78	5.4	3.3	6.0
7	Division of Labor	4.6	4.25, 4.94	1.77	5.8	0.0	6.0

## Data Availability

The study data are not publicly available in order to maintain the confidentiality of the participants.

## Supplementary Materials

The following supporting information can be downloaded at: https://www.mdpi.com/article/10.3390/children13101329/s1, Supplementary Table S1A,B: Correlation of Coparenting Relationship Scale for mothers and partners; Supplementary Table S2: Descriptive statistics for Coparenting Relationship Scale items and subscale mean scores; Supplementary Table S3: Standardized WLSMV factor loadings for CRS subscales among mothers and partners; Supplementary Table S4: Factor Loadings for Full Model Confirmatory Factor Analysis; Supplementary Table S5: Largest modification indices from the Seven-Factor Scale for partners; Supplementary Table S6: Largest modification indices from the Seven-Factor Scale for mothers; Supplementary Table S7: Cronbach’s Alpha and McDonald’s Omega Reliability Estimates for CRS Dimensions Among Mothers and Partners; Supplementary Table S8: Average Variance Extracted (AVE) for the Seven CRS Dimensions Among Mothers and Partners; Supplementary Table S9: Heterotrait-Monotrait Ratio (HTMT) Estimates for the Seven-Factor CRS Model Among Mothers and Partners.
