# Supplementary material for: Psychometric Properties of the Coparenting Relationship Scale in Primiparous Mothers and Partners in Karachi, Pakistan
Source: Children (Basel). 2026 Sep 30;13(10):1329. doi: 10.3390/children13101329 (PMC13702951; doi:10.3390/children13101329)
Supplement: Supplementary file 1 [file children-13-01329-s001.zip › children-4549215-supplementary.pdf]

Supplementary Table S1A

Correlation of Coparenting Relationship Scale for Mothers

	**Item 1**	**Item 2**	**Item 3**	**Item 4**	**Item 5**	**Item 6**	**Item 7**	**Item 8**	**Item 9**	**Item 10**	**Item11**	**Item 12**	**Item 13**	**Item 14**	**Item 15**	**Item 16**
Item 1	1	0.59	0.75	0.86	0.35	0.54	0.38	-0.41	0.23	0.55	0.25	-0.34	-0.39	0.61	0.28	-0.27
Item 2		1	0.44	0.6	0.13	0.46	0.23	-0.24	0.21	0.29	0.12	0.13	-0.09	0.47	0.07	-0.01
Item 3			1	0.66	0.26	0.55	0.38	-0.45	0.31	0.61	0.33	-0.39	-0.61	0.46	0.29	-0.31
Item 4				1	0.08	0.58	0.31	-0.3	0.19	0.38	0.09	-0.04	-0.15	0.58	0.15	-0.17
Item 5					1	0.05	0.35	-0.29	-0.01	0.2	0.11	-0.3	-0.34	0.11	0.18	-0.21
Item 6						1	029	-0.29	0.44	0.51	0.31	-0.23	-0.28	0.22	0.19	-0.1
Item 7							1	-0.41	0.48	0.43	0.51	-0.37	-0.32	0.25	0.37	-0.4
Item 8								1	-0.5	-0.21	-0.46	0.31	0.39	-0.26	-0.4	0.29
Item 9									1	0.41	0.69	-0.43	-0.31	0.06	0.53	-0.32
Item 10										1	0.44	-0.4	-0.43	0.34	0.33	-0.31
Item 11											1	-0.49	-0.29	-0.02	0.53	-0.45
Item 12												1	0.53	-0.08	-0.35	0.42
Item 13													1	-0.33	-0.52	0.52
Item 14														1	0.17	-0.15
Item 15															1	-0.51
Item 16																1

Supplementary Table S1A

Correlation of CRS for Mothers (cont'd)

	**Item 17**	**Item 18**	**Item 19**	**Item 20**	**Item 21**	**Item 22**	**Item 23**	**Item 24**	**Item 25**	**Item 26**
Item 1	0.59	0.62	0.51	0.34	-0.42	-0.31	0.63	0.64	0.5	0.67
Item 2	0.42	0.38	0.25	0.09	-0.39	-0.15	0.29	0.53	0.38	0.35
Item 3	0.62	0.46	0.53	0.31	-0.37	-0.52	0.57	0.63	0.53	0.56
Item 4	0.54	0.66	0.45	0.42	-0.33	-0.35	0.44	0.62	0.44	0.48
Item 5	0.04	0.24	0.17	0.39	-0,.24	-0.32	0.11	0.16	0.06	0.24
Item 6	0.41	0.26	0.39	0.02	-0.29	-0.23	0.33	0.37	0.3	0.3
Item 7	0.1	0.45	0.51	0.35	-0.3	-0.54	0.33	0.31	0.43	0.45
Item 8	-0.22	-0.34	-0.45	-0.37	0.24	0.35	-0.34	-0.33	-0.32	-0.45
Item 9	0.17	0.19	0.43	0.13	-0.2	-0.37	0.22	0.3	0.45	0.43
Item 10	0.34	0.45	0.22	0.15	-0.21	-0.31	0.4	0.53	0.62	0.52
Item 11	0.15	0.28	0.12	0.19	-0.23	-0.33	0.26	0.2	0.34	0.4
Item 12	-0.21	-0.18	-0.2	-0.23	0.25	0.42	-0.32	-0.11	-0.45	-0.46
Item 13	-0.45	-0.31	-0.42	-0.33	0.24	0.57	-0.37	-0.36	-0.54	-0.49
Item 14	0.45	0.47	0.48	0.19	-0.32	-0.36	0.39	0.63	0.47	0.44
Item 15	0.31	0.27	0.27	0.26	-0.34	-0.5	0.37	0.25	0.3	0.4
Item 16	-0.42	-0.4	-0.31	-0.31	0.3	0.48	-0.28	-0.32	-0.54	-0.56
Item 17	1	0.5	0.4	0.28	-0.47	-0.41	0.47	0.68	0.47	0.51
Item 18		1	0.39	0.35	-0.19	-0.26	0.42	0.64	0.53	0.59
Item 19			1	0.38	-0.25	-0.52	0.49	0.52	0.6	0.68
Item 20				1	-0.26	-0.48	0.42	0.31	0.25	0.34
Item 21					1	0.47	-0.37	-0.45	-0.31	-0.41
Item 22						1	-0.38	-0.46	-0.56	-0.56
Item 23							1	0.67	0.46	0.56
Item 24								1	0.68	0.6
Item 25									1	
Item 26										1
Item 27										
Item 28										
Item 29										
Item 30										
Item 31										
Item 32										
Item 33										
Item 34										
Item 35										

Supplementary Table S1A

Correlation of CRS for Mothers (cont'd)

	**Item 27**	**Item 28**	**Item 29**	**Item 30**	**Item 31**	**Item 32**	**Item 33**	**Tem 34**	**Item 35**
Item 1	0.52	0.27	0.41	0.63	-0.49	-0.51	-0.51	-0.6	-0.57
Item 2	0.35	0.36	0.25	0.37	-0.31	-0.39	-0.,28	-0.33	-0.19
Item 3	0.6	0.44	0.37	0.61	-0.35	-0.54	-0.5	-0.54	-0.48
Item 4	0.5	0.31	0.37	0.55	-0.26	-0.4	-0.34	-0.49	-0.49
Item 5	0.17	0.04	0.17	0.18	-0.09	-0.08	-0.35	-0.24	-0.25
Item 6	0.49	0.25	0.15	0.37	-0.38	-0.33	-0.23	-0.39	-0.22
Item 7	0.44	0.41	0.29	0.21	-0.24	-0.32	-0.19	-0.26	-0.29
Item 8	-0.28	-0.49	-0.38	-0.27	0.24	0.27	0.08	0.26	0.3
Item 9	0.5	0.46	0.19	0.2	-0.31	-0.31	-0.02	-0.23	-0.17
Item 10	0.61	0.23	0.17	0.33	-0.3	-0.42	-0.42	-0.39	-0.42
Item 11	0.32	0.44	0.16	0.02	-0.25	-0.31	-0.22	-0.18	-0.19
Item 12	-0.4	-0.24	-0.28	-0.12	0.33	0.5	0.35	0.32	0.28
Item 13	-0.54	-0.34	-0.31	-0.24	0.27	0.49	0.41	0.47	0.39
Item 14	0.4	0.29	0.21	0.58	-0.28	-0.37	-0.37	-0.42	-0.44
Item 15	0.37	0.37	0.36	0.21	-0.31	-0.27	-0.16	-0.32	-0.4
Item 16	-0.29	-0.47	-0.3	-0.27	0.14	0.45	0.36	0.5	0.45
Item 17	0.42	0.43	0.35	0.59	-0.34	-0.44	-0.38	-0.47	-0.39
Item 18	0.46	0.32	0.26	0.47	-0.34	-0.49	-0.45	-0.56	-0.49
Item 19	0.52	0.32	0.17	0.6	-0.23	-0.37	-0.13	-0.42	-0.28
Item 20	0,.24	0.,38	0.43	0.23	-0.22	-0.21	-0.22	-0.3	-0.45
Item 21	-0.41	-0.39	-0.36	-0.41	0.32	0.27	0.38	0.29	0.25
Item 22	-0.51	-0.52	-0.53	-0.38	0.25	0.42	0.34	0.43	0.34
Item 23	0.59	0.29	0.37	0.64	-0.28	-0.41	-0.42	-0.51	-0.4
Item 24	0.62	0.46	0.47	0.78	-0.39	-0.46	-0.44	-0.42	-0.37
Item 25	0.73	0.42	0.35	0.52	-0.27	-0.63	-0.48	-0.41	-0.43
Item 26	0.62	0.47	0.34	0.63	-0.28	-0.49	-0.4	-0.51	-0.33
Item 27	1	0.24	0.21	0.63	-0.34	-0.53	-0.48	-0.47	-0.29
Item 28		1	0.55	0.33	-0.41	-0.34	-0.19	-0.36	-0.44
Item 29			1	0.27	-0.34	-0.37	-0.32	-0.21	-0.39
Item 30				1	-0.35	-0.23	-0.32	-0.46	-0.21
Item 31					1	0.56	0.46	0.31	0.39
Item 32						1	0.66	0.62	0.59
Item 33							1	0.71	0.67
Item 34								1	0.76
Item 35									1

Supplementary Table S1B

Correlation of Coparenting Relationship Scale for Partners

	Item 1	Item 2	Item 3	Item 4	Item 5	Item 6	Item 7	Item 8	Item 9	Item 10	Item 11	Item 12	Item 13	Item 14	Item 15	Item 16
Item 1	1	0.66	0.44	0.67	0.02	0.57	0.38	-0.25	0.19	0.44	0.33	-0.47	-0.5	0.44	0.32	-0.55
Item 2		1	0.55	0.69	0.07	0.36	0.27	-0.24	0.02	0.33	0.31	-0.36	-0.58	0.32	0.37	-0.43
Item 3			1	0.69	0.01	0.35	0.28	-0.33	0.36	0.33	0.,26	-0.19	-0.33	0.26	0.36	-0.31
Item 4				1	0.33	0.43	0.49	-0.39	0.35	0.3	0.39	-0.22	-0.55	0.38	0.49	-0.43
Item 5					1	-0.09	0.41	-0.44	0.21	0.09	0.35	-0.2	-0.43	0.14	0.49	-0.1
Item 6						1	0.34	-0.21	0.23	0.42	0.28	-0.21	-0.14	0.19	0.21	-0.22
Item 7							1	-0.57	0.41	0.,25	0.39	-0.51	-0.53	0.,21	0.4	-0.47
Item 8								1	-0.46	-0.19	-0.55	0.27	0.43	0.06	-0.58	0.41
Item 9									1	0.28	0.65	-0.4	-0.28	0.08	0.62	-0.51
Item 10										1	0.22	-0.19	-0.46	0.33	0.22	-0.36
Item 11											1	-0.48	-0.43	-0.03	0.,74	-0.61
Item 12												1	0.48	-0.09	-0.34	0.51
Item 13													1	-0.5	-0.43	0.67
Item 14														1	0.02	-0.16
Item 15															1	-0.52
Item 16																1

Supplementary Table S1B

Correlation of Coparenting Relationship Scale for Partners (cont'd)

	Item 17	Item 18	Item 19	Item 20	Item 21	Item 22	Item 23	Item 24	Item 25	Item 26
Item 1	0.5	0.5	0.34	0.3	-0.15	-0.45	0.39	0.53	0.54	0.56
Item 2	0.46	0.25	0.64	0.36	-0.1	-0.4	0.46	0.48	0.6	0.54
Item 3	0.,27	0.16	0.44	0.22	-0.14	-0.21	0.3	0.37	0.48	0.24
Item 4	0.44	0.36	0.53	0.45	-0.08	-0.36	0.45	0.44	0.41	0.41
Item 5	0.12	0.22	-0.06	0.61	-0.33	-0.43	0.26	0.31	-0.01	0.11
Item 6	0.39	0.41	0.25	0.26	-0.16	-0.2	0.34	0.46	0.48	0.36
Item 7	0.31	0.35	0.21	0.53	-0.37	-0.53	0.42	0.37	0.39	0.4
Item 8	-0.28	-0.22	-0.04	-0.48	0.36	0.47	-0.32	-0.22	-0.09	-0.24
Item 9	0.,12	0.12	0.21	0.49	-0.43	-0.42	0.18	0.25	0.18	0.28
Item 10	0.27	0.17	0.33	0.09	-0.1	-0.39	0.41	0.6	0.62	0.5
Item 11	0.,15	0.25	0.28	0.52	-0.4	-0.41	0.19	0.31	0.26	0.31
Item 12	-0.08	-0.13	-0.12	-0.41	0.52	0.58	-0.25	-0.37	-0.33	-0.29
Item 13	-0.33	-0.17	-0.31	-0.54	0.2	0.67	-0.21	-0.53	-0.46	-0.49
Item 14	0.27	0.08	0.22	0.29	-0.02	-0.31	0.34	0.44	0.45	0.46
Item 15	0.44	0.28	0.41	0.54	-0.34	-0.35	0.22	0.39	0.26	0.41
Item 16	-0.26	-0.25	-0.39	-0.48	0.46	0.64	-0.19	-0.41	-0.36	-0.44
Item 17	1	0.6	0.49	0.45	-0.19	-0.23	0.54	0.56	0.58	0.6
Item 18		1	0.26	0.37	-0.11	-0.17	0.35	0.39	0,.36	0.51
Item 19			1	0.38	-0.15	-0.38	0.47	0.59	0.65	0.66
Item 20				1	-0.66	-0.72	0.52	0.69	0.47	0.65
Item 21					1	0.6	-0.27	-0.26	-0.25	-0.32
Item 22						1	-0.43	-0.57	-0.49	-0.51
Item 23							1	0.75	0.72	0.67
Item 24								1	0.85	0.82
Item 25									1	0.86
Item 26										1
Item 27										
Item 28										
Item 29										
Item 30										
Item 31										
Item 32										
Item 33										
Item 34										
Item 35										

Supplementary Table S1B

Correlation of Coparenting Relationship Scale for Partners (cont'd)

	Item 27	Item 28	Item 29	Item 30	Item 31	Item 32	Item 33	Item 34	Item 35
Item 1	0.32	0.23	0.22	0.38	-0.3	-0.19	-0.27	-0.23	-0.24
Item 2	0.53	0.26	0.16	0.44	-0.35	-0.28	-0.14	-0.09	-0.24
Item 3	0.35	0.27	0.05	0.28	-0.27	-0.4	-0.2	-0.2	-0.27
Item 4	0.41	0.25	0.32	0.34	-0.25	-0.32	-0.32	-0.42	-0.45
Item 5	0.19	0.22	0.53	0.11	-0.25	-0.14	-0.37	-0.33	-0.19
Item 6	0.42	0.36	0.24	0.26	-0.27	-0.12	-0.28	-0.39	-0.32
Item 7	0.36	0.49	0.46	0.29	-0.29	-0.33	-0.38	-0.43	-0.34
Item 8	-0.04	-0.57	-0.44	-0.14	0.36	0.3	0.34	0.45	0.44
Item 9	0.19	0.48	0.4	0.15	-0.44	-0.36	-0.31	-0.33	-0.27
Item 10	0.49	0.22	0.27	0.31	-0.28	-0.3	-0.28	-0.26	-0.37
Item 11	0.18	0.56	0.53	0.17	-0.56	-0.4	-0.46	-0.53	-0.45
Item 12	-0.26	-0.31	-0.35	-0.11	0.25	0.25	0.2	0.16	0.15
Item 13	-0.45	-0.37	-0.46	-0.29	0.22	0.34	0.36	0.43	0.46
Item 14	0.48	-0.01	0.1	0.2	-0.14	-0.12	-0.14	-0.04	-0.14
Item 15	0.28	0.5	0.53	0.25	-0.44	-0.39	-0.4	-0.47	-0.31
Item 16	-0.22	-0.48	-0.47	-0.2	0.29	0.53	0.34	0.42	0.38
Item 17	0.49	0.41	0.28	0.57	-0.4	-0.33	-0.39	-0.42	-0.5
Item 18	0.34	0.53	0.5	0.26	-0.36	-0.24	-0.28	-0.28	-0.34
Item 19	0.53	0.17	0.27	0.61	-0.33	-0.55	-0.44	-0.34	-0.44
Item 20	0.5	0.58	0.6	0.4	-0.58	-0.49	-0.66	-0.64	-0.58
Item 21	-0.21	-0.32	-0.39	-0.22	0.45	0.37	0.41	0.42	0.34
Item 22	-0.55	-0.45	-0.56	-0.34	0.41	0.44	0.54	0.52	0.51
Item 23	0.62	0.28	0.23	0.59	-0.5	-0.36	-0.45	-0.39	-0.44
Item 24	0.85	0.34	0.45	0.62	-0.41	-0.4	-0.56	-0.46	-0.42
Item 25	0.88	0.33	0.3	0.59	-0.4	-0.27	-0.4	-0.29	-0.39
Item 26	0.77	0.36	0.4	0.62	-0.51	-0.43	-0.54	-0.51	-0.48
Item 27	1	0.25	0.33	0.56	-0.32	-0.21	-0.36	-0.27	-0.28
Item 28		1	0.61	0.07	-0.45	-0.38	-0.35	-0.46	-0.49
Item 29			1	0.35	-0.4	-0.32	-0.45	-0.52	-0.55
Item 30				1	-0.26	-0.29	-0.35	-0.42	-0.4
Item 31					1	0.7	0.69	0.59	0.57
Item 32						1	0.72	0.69	0.7
Item 33							1	0.88	0.82
Item 34								1	0.93
Item 35									1

Supplementary Table S2

Descriptive Statistics for Coparenting Relationship Scale Items and Subscale Mean Scores

Item	Mean	SD	Median	Floor (Min) %	Ceiling (Max) %	Mean	SD	Median	Floor (Min) %	Ceiling (Max) %
			Mothers					Partners		
CRS01	5.6	1.1	6	0.8	78.3	5.7	0.7	6	0.0	82.4
CRS02	5.1	1.3	6	1.7	54.2	5.4	1.1	6	0.0	65.7
CRS03	5.3	1.3	6	2.5	65.0	5.3	1.3	6	2.0	63.7
CRS04	5.5	1.0	6	0.8	71.7	5.7	0.7	6	0.0	80.4
CRS05	2.8	2.5	2	31.7	26.7	4.1	2.5	6	19.6	54.9
CRS06	5.1	1.5	6	2.5	60.0	5.2	1.3	6	2.0	54.9
CRS07	4.6	2.0	6	10.8	54.2	4.9	1.7	6	3.9	62.7
CRS08	1.3	2.0	0	64.2	7.5	1.3	2.0	0	59.8	7.8
CRS09	4.4	2.0	5	6.7	45.0	3.8	2.1	5	6.9	33.3
CRS10	4.6	1.8	5	6.7	47.5	4.8	1.6	5	4.9	44.1
CRS11	4.0	2.2	5	10.0	41.7	4.1	2.1	5	5.9	44.1
CRS12	1.5	2.0	0	54.2	9.2	1.9	2.1	1	39.2	7.8
CRS13	0.5	1.2	0	77.5	1.7	0.9	1.6	0	69.6	2.9
CRS14	5.0	1.7	6	5.8	60.8	5.0	1.8	6	7.8	62.7
CRS15	4.5	1.9	5	3.3	48.3	4.3	2.0	5	4.9	45.1
CRS16	1.2	1.9	0	59.2	7.5	1.9	2.3	1	49.0	14.7
CRS17	5.5	1.2	6	1.7	74.2	5.3	1.3	6	2.0	67.6
CRS18	5.3	1.3	6	1.7	70.8	5.1	1.5	6	2.0	59.8
CRS19	5.2	1.5	6	2.5	65.0	5.3	1.1	6	0.0	60.8
CRS20	4.7	1.8	6	3.3	53.3	5.1	1.7	6	4.9	71.6
CRS21	1.7	2.1	0	50.8	5.8	1.7	2.1	0	52.0	5.9
CRS22	0.9	1.7	0	66.7	5.8	1.1	1.8	0	65.7	3.9
CRS23	4.9	1.7	6	2.5	56.7	5.2	1.3	6	2.0	61.8
CRS24	5.4	1.2	6	1.7	70.8	5.5	1.0	6	1.0	73.5
CRS25	5.1	1.5	6	2.5	62.5	5.3	1.2	6	1.0	63.7
CRS26	5.3	1.3	6	1.7	65.0	5.4	1.1	6	1.0	65.7
CRS27	5.0	1.5	6	4.2	54.2	5.2	1.4	6	2.0	63.7
CRS28	4.5	2.0	6	6.7	55.0	4.6	2.0	6	5.9	56.9
CRS29	5.0	1.7	6	5.8	62.5	4.9	1.9	6	5.9	68.6
CRS30	5.4	1.1	6	0.8	64.2	5.5	0.9	6	0.0	70.6
CRS31	1.3	1.5	1	40.0	3.3	1.1	1.5	0	52.0	2.0
CRS32	1.1	1.5	0	50.8	1.7	1.2	1.7	0	53.9	3.9
CRS33	0.8	1.5	0	64.2	2.5	0.7	1.3	0	66.7	1.0
CRS34	0.6	1.3	0	73.3	1.7	0.6	1.2	0	70.6	0.0
CRS35	0.6	1.3	0	76.7	1.7	0.5	1.1	0	76.5	0.0

Floor effects represent the proportion of respondents selecting the minimum score (0), and ceiling effects represent the proportion selecting the maximum score (6). Among mothers, 11 items showed floor effects exceeding 50%, with the highest observed for Item 13 (77.5%). Partners showed a similar pattern, with 10 items exceeding the 50% floor threshold, and a maximum of 76.5% for Item 35. Ceiling effects were more pronounced: 19 items exceeded 50% among mothers (maximum 78.3%, Item 1), and 20 items exceeded 50% among partners (maximum 82.4%, Item 1). These findings indicate considerable response clustering at scale extremes, particularly for items reflecting closeness and support.

Supplementary Table S3

Standardized WLSMV Factors Loadings for Coparenting Relationship Subscale among Mothers and Partners

**Factors**	**Item**	**Mothers**	**Partners**
Childrearing Agreement	CRS06	*0.44*	*0.30*
Childrearing Agreement	CRS09	**0.88**	0.74
Childrearing Agreement	CRS11	0.79	**0.89**
Childrearing Agreement	CRS15	0.62	0.83
Coparenting Closeness	CRS02	0.55	0.61
Coparenting Closeness	CRS17	0.74	0.76
Coparenting Closeness	CRS24	**0.96**	0.79
Coparenting Closeness	CRS28	0.50	*0.41*
Coparenting Closeness	CRS30	0.79	0.74
Exposure to Conflict	CRS31	0.55	0.76
Exposure to Conflict	CRS32	0.80	0.79
Exposure to Conflict	CRS33	0.83	**0.90**
Exposure to Conflict	CRS34	0.84	**0.97**
Exposure to Conflict	CRS35	0.83	0.94
Endorsement of Partners Parenting	CRS01	**0.96**	0.78
Endorsement of Partners Parenting	CRS04	**0.88**	0.75
Endorsement of Partners Parenting	CRS07	*0.46*	0.67
Endorsement of Partners Parenting	CRS14	0.64	*0.42*
Endorsement of Partners Parenting	CRS18	0.72	0.59
Endorsement of Partners Parenting	CRS23	0.61	0.59
Endorsement of Partners Parenting	CRS29	*0.44*	0.56
Coparental Support	CRS03	0.71	*0.46*
Coparental Support	CRS10	0.68	0.59
Coparental Support	CRS19	0.68	0.68
Coparental Support	CRS25	**0.92**	**1.00**
Coparental Support	CRS26	**0.88**	**0.87**
Coparental Support	CRS27	0.79	**0.87**
Coparenting Undermining	CRS08	*0.48*	0.53
Coparenting Undermining	CRS12	0.61	0.67
Coparenting Undermining	CRS13	0.76	0.74
Coparenting Undermining	CRS16	0.66	0.79
Coparenting Undermining	CRS21	0.48	0.61
Coparenting Undermining	CRS22	0.76	**0.89**

CRS Coparenting Relationship Scale. Standardized factor loadings reflect the strength of the association between each item and its underlying latent factor. Higher loadings indicate stronger relationships and better item representation of the construct. Loadings ≥ 0.50 are considered acceptable, loadings < 0.40 indicate weak item–factor alignment, while scores between 0.4 and 0.5 indicate marginal association.

Supplementary Table S4

Factor Loadings for Full Model Confirmatory Factor Analysis

**Factors**	**Item**	**Mothers**	**Partners**
Childrearing Agreement	CRS06	0.72	0.58
Childrearing Agreement	CRS09	0.76	0.67
Childrearing Agreement	CRS11	0.70	0.82
Childrearing Agreement	CRS15	0.71	0.83
Coparenting Closeness	CRS02	0.56	0.65
Coparenting Closeness	CRS17	0.75	0.63
Coparenting Closeness	CRS24	**0.90**	0.85
Coparenting Closeness	CRS28	0.63	0.58
Coparenting Closeness	CRS30	0.78	0.60
Exposure to Conflict	CRS31	0.61	0.82
Exposure to Conflict	CRS32	0.84	0.79
Exposure to Conflict	CRS33	0.76	0.91
Exposure to Conflict	CRS34	0.86	0.96
Exposure to Conflict	CRS35	0.79	0.94
Endorsement of Partners Parenting	CRS01	**0.92**	0.67
Endorsement of Partners Parenting	CRS04	0.79	0.68
Endorsement of Partners Parenting	CRS07	0.61	0.64
Endorsement of Partners Parenting	CRS14	0.65	*0.40*
Endorsement of Partners Parenting	CRS18	0.73	0.52
Endorsement of Partners Parenting	CRS23	0.74	0.70
Endorsement of Partners Parenting	CRS29	0.55	0.66
Division of Labor	CRS05	*0.49*	0.55
Division of Labor	CRS10	0.78	**1.10**
Coparental Support	CRS03	0.84	0.57
Coparental Support	CRS10	0.68	0.59
Coparental Support	CRS19	0.70	0.73
Coparental Support	CRS25	0.86	0.93
Coparental Support	CRS26	0.87	0.93
Coparental Support	CRS27	0.80	0.86
Coparenting Undermining	CRS08	0.59	0.65
Coparenting Undermining	CRS12	0.56	0.59
Coparenting Undermining	CRS13	0.71	0.78
Coparenting Undermining	CRS16	0.62	0.76
Coparenting Undermining	CRS21	0.57	0.60
Coparenting Undermining	CRS22	0.74	0.86

Loadings ≥ 0.50 are considered acceptable, loadings < 0.40 indicate weak item–factor alignment, and values between 0.40 and 0.50 reflect marginal association. In this table, all mother loadings met or exceeded the acceptable threshold, whereas partners showed one weak loading (CRS14 = 0.40) and several marginal loadings (e.g., CRS6 = 0.58, CRS28 = 0.58), indicating slightly lower item–factor coherence for partners relative to mothers.

Supplementary Table S5

Largest Modifications Indices from the Seven-Factor Scale for Partners

LHS	RHS	mi	Sepc.all
Coparental Support	CRS02	2819.89	26.62
Endorsement of Partner Parenting	CRS02	1238.57	12.21
Coparental Support	CRS24	161.56	0.69
CRS01	CRS01	125.23	1.00
Endorsement of Partner Parenting	CRS01	125.23	5.73
CRS24	CRS24	124.18	1.00
Coparenting Closeness	CRS01	118.77	5.26
CRS03	CRS03	106.01	1.00
Coparental Support	CRS03	106.01	6.39
Endorsement of Partner Parenting	CRS24	105.06	0.56
Childrearing Agreement	CRS28	82.50	0.61
Coparenting Undermining	CRS28	72.85	-0.56
Coparental Support	CRS06	72.48	0.62
Coparenting Closeness	CRS06	69.72	0.79
Endorsement of Partner Parenting	CRS06	66.70	0.92
Division of Labor	CRS28	61.76	0.55
Coparenting Undermining	CRS02	54.20	-0.69
Coparenting Closeness	CRS26	49.73	0.51
Exposure to Conflict	CRS28	48.00	-0.46
CRS25	CRS25	47.76	1.00
Coparental Support	CRS23	44.97	0.52
Endorsement of Partner Parenting	CRS28	44.03	0.47
Coparenting Undermining	CRS07	43.85	-0.62
Endorsement of Partner Parenting	CRS26	42.79	0.44
Coparenting Undermining	CRS24	41.39	-0.36
Division of Labor	CRS26	40.05	0.37
CRS26	CRS26	39.00	1.00
Division of Labor	CRS24	38.69	0.38
Exposure to Conflict	CRS24	36.54	-0.35
Coparenting Closeness	CRS03	33.70	1.09

RHS = Right hand side; LHS = Left Hand Side; CRS Coparenting Relationship Scale. Largest modification indices (mi) identify parameters whose freeing would most substantially improve model fit, while SEPC.all reflects the expected standardized change in that parameter if freed. In this table, the largest values (e.g., MI = 2819.89 and SEPC.all = 26.62 for Coparental Support → CRS02, and MI = 1238.57 with SEPC.all = 12.21 for Endorsement of Partner Parenting → CRS02) indicate pronounced cross-factor associations suggesting areas of notable local misfit. Rows where the LHS and RHS refer to the same item (e.g., CRS01 ↔ CRS01 with MI = 125.23 and SEPC.all = 1.00) indicate that allowing the item’s residual variance to adjust would meaningfully reduce misfit.

Supplementary Table S6

Largest Modifications Indices from the Seven-Factor Scale for Mothers

LHS	RHS	Mi	Sepc.all
Coparenting Undermining	CRS07	52.15	-0.82
Childrearing Agreement	CRS07	47.46	0.61
Coparenting Closeness	CRS06	45.15	0.60
Coparenting Support	CRS06	43.57	0.77
Endorsement of Partner Parenting	CRS06	41.73	0.55
Coparenting Undermining	CRS28	39.95	-0.66
Coparenting Agreement	CRS28	33.89	0.52
Coparental Support	CRS07	32.42	0.98
Coparenting Undermining	CRS04	23.53	0.52
CRS01	CRS04	23.13	1.10
Exposure to Conflict	CRS06	22.93	-0.43
Coparental Support	CRS28	18.96	0.81
CRS09	CRS11	16.67	0.63
Division of Labor	CRS28	16.56	0.43
Coparenting Closeness	CRS11	16.00	-0.34
Coparenting Closeness	CRS21	15.76	-0.44
Coparental Support	CRS11	15.57	-0.44
Coparental Support	CRS04	15.53	-0.63
Coparenting Undermining	CRS06	14.03	-0.62
Endorsement of Partners Parenting	CRS11	13.53	-0.30
Endorsement of Partner Parenting	CRS21	13.42	-0.43
CRS11	CRS07	13.41	0.53
Coparenting Closeness	CRS12	13.35	0.42
CRS06	CRS04	13.08	0.71
Childrearing Agreement	CRS04	12.98	-0.31
Expoure to Conflict	CRS28	12.57	-0.39
CRS02	CRS12	11.47	0.52
Division of Labor	CRS29	11.00	0.41
CRS06	CRS15	10.98	-0.77
Exposure to Conflict	CRS09	10.81	0.32

Note: RHS = Right hand side; LHS = Left Hand Side, CRS=Coparenting Relationship Scale. For mothers, the largest MI and SEPC.all values were modest (e.g., Coparenting Undermining → CRS07, MI = 52.15, SEPC.all = –0.82; CRS01 ↔ CRS04, MI = 23.13, SEPC.all = 1.10), indicating largest mi falls well below conventional thresholds (mi>100, |SPEC.all|>1) for substantial misfit, indicating that the seven-factor structure provides an adequate representation of item–factor relations with only minor residual associations.

Supplementary Table S7

Cronbach's Alpha and McDonald's Omega Reliability Estimates for CRS Dimensions Among Mothers and Partners

CRS Subscale	No. of Items	Mothers Cronbach's α	Mothers McDonald's ω	Partners Cronbach's α	Partners McDonald's ω
Agreement	4	0.70	0.76	0.74	0.80
Closeness	5	0.71	0.71	0.56	0.66
Conflict	5	0.82	0.83	0.88	0.93
Support	6	0.85	0.88	0.78	0.85
Undermining	6	0.66	0.70	0.79	0.81
Endorsement	7	0.73	0.74	0.59	0.70
Division of Labour	2	N/A*			

Note. α = Cronbach's alpha; ω = McDonald's omega. Higher values indicate greater internal consistency, with values around 0.70 or higher considered indicative of acceptable reliability. McDonald's ω was used as the primary reliability estimate because it allows factor loadings to vary across items, while Cronbach's α was retained to facilitate comparison with previous CRS studies. Reliability coefficients were not reported for the two-item Division of Labor dimension using the same procedure applied to the multi-item dimensions. Its psychometric performance was instead evaluated within the full seven-factor CFA using standardized factor loadings and AVE.

Internal consistency estimates varied across CRS dimensions and between mothers and partners. Among mothers, McDonald's ω ranged from 0.70 for Coparenting Undermining to 0.88 for Coparental Support, with the remaining dimensions ranging from 0.71 to 0.83. Among partners, ω ranged from 0.66 for Coparenting Closeness to 0.93 for Exposure to Conflict. Coparenting Agreement (ω = 0.80), Coparental Support (ω = 0.85), and Coparenting Undermining (ω = 0.81) also demonstrated good internal consistency among partners, whereas Endorsement of Partner's Parenting was lower (ω = 0.70). Cronbach's α estimates were generally consistent with, but somewhat lower than, the corresponding ω estimates, particularly among partners.

As discussed in the manuscript, Division of Labor comprised only two items, hence its psychometric performance was evaluated within the full seven-factor CFA rather than using the same reliability procedure applied to the multi-item dimensions. A standalone congeneric model was underidentified (df = −1), while imposing tau-equivalent loadings resulted in a saturated model (df = 0), precluding an informative chi-square difference test. Consequently, standardized factor loadings and average variance extracted (AVE) from the identified seven-factor CFA were used as supplementary evidence for this dimension (Supplementary Table 8).

Supplementary Table S8

Average Variance Extracted (AVE) for the Seven CRS Dimensions Among Mothers and Partners

**CRS dimension**	**Mothers AVE**	**Partners AVE**
Agreement	**0.52**	**0.54**
Closeness	**0.54**	0.45
Conflict	**0.61**	**0.78**
Support	**0.63**	**0.62**
Undermining	0.40	**0.51**
Endorsement	**0.52**	0.38
Division of Labor	0.43	**0.76**

AVE estimates ranged from 0.40 to 0.63 among mothers and from 0.38 to 0.78 among partners. Five of the seven dimensions achieved AVE ≥0.50 in each group. Among mothers, AVE was below 0.50 for Coparenting Undermining (0.40) and Division of Labor (0.43), whereas among partners, lower AVE values were observed for Coparenting Closeness (0.45) and Endorsement of Partner's Parenting (0.38).

Discriminant validity was further assessed using the heterotrait-monotrait ratio (HTMT), with results presented in Supplementary Table 9. Among mothers, most HTMT values were below 0.85, although elevated values were observed for Coparenting Closeness-Endorsement of Partner's Parenting (0.93), Coparental Support-Endorsement of Partner's Parenting (0.91), Coparental Support-Coparenting Undermining (0.86), and Coparenting Closeness-Coparental Support (0.85). These findings indicate some overlap among dimensions, particularly Closeness, Support, and Endorsement, although the seven-factor model for mothers remained admissible. Among partners, particularly high HTMT values were observed for Coparenting Closeness-Coparental Support (1.03) and Coparenting Closeness-Endorsement of Partner's Parenting (0.98). These findings indicate greater concerns regarding discriminant validity among these dimensions for partners than for mothers.

Supplementary Table S9

Heterotrait-Monotrait Ratio (HTMT) Estimates for the Seven-Factor CRS Model Among Mothers and Partners

**CRS Dimension**	**AGR**	**CLS**	**CON**	**SUP**	**UND**	**END**	**DOL**
**Mother**							
Agreement (AGR)	1						
Closeness (CLS)	0.49	1					
Conflict (CON)	0.48	0.67	1				
Support (SUP)	0.75	**0.85**	0.7	1			
Undermining (UND)	0.84	0.61	0.7	**0.86**	1		
Endorsement (END)	0.54	**0.93**	0.79	**0.91**	0.74	1	
Division of Labor (DOL)	0.21	0.33	0.47	0.46	0.79	0.61	1
**Partners**							
**CRS Dimension**	**AGR**	**CLS**	**CON**	**SUP**	**UND**	**END**	**DOL**
Agreement (AGR)	1						
Closeness (CLS)	0.77	1					
Conflict (CON)	0.67	0.68	1				
Support (SUP)	0.64	**1.03**	0.56	1			
Undermining (UND)	0.82	0.69	0.61	0.52	1		
Endorsement (END)	0.69	**0.98**	0.6	**0.86**	0.71	1	
Division of Labor (DOL)	0.65	0.55	0.57	0.21	0.75	0.65	1

Bold values indicate HTMT ≥ 0.85 and therefore factor pairs warranting closer examination for discriminant validity. *HTMT values should be interpreted alongside the latent factor correlations and other CFA diagnostics rather than as a standalone criterion.*
